# Animal Health in Compost-Bedded Pack and Cubicle Dairy Barns in Six European Countries

**DOI:** 10.3390/ani12030396

**Published:** 2022-02-07

**Authors:** Ulf Emanuelson, Kerstin Brügemann, Marija Klopčič, Lorenzo Leso, Wijbrand Ouweltjes, Andreas Zentner, Isabel Blanco-Penedo

**Affiliations:** 1 Department of Clinical Sciences, Swedish University of Agricultural Sciences, SE-75007 Uppsala, Sweden; isabel.blanco.penedo@slu.se; 2 Institute of Animal Breeding and Genetics, Justus-Liebig-University, D-35390 Giessen, Germany; Kerstin.Bruegemann@agrar.uni-giessen.de; 3 Department of Animal Science, Biotechnical Faculty, University of Ljubljana, Groblje 3, 1230 Domžale, Slovenia; Marija.Klopcic@bf.uni-lj.si; 4 Department of Agricultural, Food and Forestry Systems, University of Florence, 50145 Florence, Italy; lorenzo.leso@unifi.it; 5 Wageningen Livestock Research, P.O. Box 338, 6700 AH Wageningen, The Netherlands; wijbrand.ouweltjes@wur.nl; 6 HBLFA Raumberg-Gumpenstein, 8952 Irdning-Donnersbachtal, Austria; Andreas.Zentner@raumberg-gumpenstein.at

**Keywords:** dairy cattle, housing system, management, health traits, disease incidence, mastitis, longevity

## Abstract

**Simple Summary:**

Dairy barns with compost-bedded pack housing systems are relatively new in Europe. They have housing systems that are vastly different from traditional tie-stall or cubicle housing and provide possibilities for improved animal welfare. However, it is important to investigate how actual cow health is influenced. We used readily available data from 32 dairy herds throughout Europe, half of which had compost-bedded pack housing and half cubicle housing, to investigate differences in dairy cow health. We found that herds with compost-bedded packs had poorer udder health than did herds with cubicles, while they seemed to have fewer problems with reproductive disorders. Our conclusion was that there were few, relatively minor differences between the systems. This knowledge is valuable for farmers interested in applying new housing systems for dairy cows and for consumers who want to stay informed about production conditions in dairy herds.

**Abstract:**

The purpose of this study was to compare animal health in compost-bedded pack (CBP) and cubicle housing (CH) systems using data from dairy herd improvement associations. Thirty-two commercial dairy farms located in Austria, Germany, Italy, The Netherlands, Slovenia, and Sweden were included in the study. A matching design (pairing CBP and CH within country) according to herd selection criteria was used. We explored the following health indicators: somatic cell counts (SCC), high SCC, new high SCC, ketosis risk, prolonged calving intervals, dystocia, and stillbirth. Traits for culling and culling-related issues, such as length of life and length of productive life, were also included. We used multivariable (mixed) linear and logistic regression models to evaluate differences between the systems. Udder health, as measured by SCC, was inferior in CBP, although the geometric means were low in both systems. The incidence of stillbirths was higher in CBP, while prolonged calving intervals were fewer, indicating that there were fewer reproductive disorders. There were no differences in longevity between the systems, although CBP had lower proportions of first calvers. Overall, we conclude that there were few and minor differences in health and longevity between the CBP and CH systems in the European context.

## 1. Introduction

Animal housing affects the physical, physiological, and behavioral aspects of farm animals, so it has an impact on their health and welfare [1]. The design of housing facilities and management practices are also important elements of future farm systems because they affect sustainability aspects, such as the environment, cattle health and welfare, and economics [2]. One last, and increasingly important, aspect to be considered regarding animal housing is the societal demand to increase animal welfare [3,4], although a consumer study in eight European countries did not show a higher marketing value for a particular housing system [5].

Recent reports in the EU, based on official audits and a literature review [6,7], show that housing systems are major factors influencing animal welfare and that the welfare of dairy cows is insufficiently monitored. They show that lameness, mastitis, metabolic diseases, and reproductive problems remain critical areas, confirming that there are major animal health and welfare issues across different dairy production systems. The ongoing challenges associated with high rates of lameness in cows are of great concern for the global dairy sector [3]. The most important issues to be improved compromise of cleanliness, which has been linked to mastitis [8,9], and a high prevalence of claw lesions in dairy cows [10].

Therefore, a challenge in future housing involves creating designs that resolve conflicts in existing housing systems. In this regard, new housing systems have to be considered and developed. Compost-bedded pack (CBP) barns are promising housing systems from the point of view of animal welfare and the environment. Similarly, to straw yards, in CBP, cows are provided with open-bedded pack areas for resting and exercise where cow excreta is mixed with bedding materials. However, the entire pack in CBP is cultivated one to three times per day, aerated by blowing or suckling air, and the area per cow required is generally higher than that in other housing systems [2]. Dairy producers mentioned that the main reasons for building CBP systems are to improve cow comfort, cow longevity, and the ease with which animals meet their daily needs [11,12].

So far, few studies have compared dairy cattle health in CBP versus cubicle housing (CH) systems. A recent review [12] found that CBP systems have the potential to improve the welfare of dairy cows. Other studies reported that cattle in CBP barns had better foot and leg health than did cattle in CH barns, as indicated by reduced lameness and hock lesion prevalence [10]. Endres and Barberg [13] reported that “cow foot and leg health” were good in the CBPs visited. Eckelkamp et al. [14,15] concluded that, when managed according to recommendations, CBP reduced lameness, improved hock health, reduced SCC, maintained cow cleanliness, and increased milk production when compared with CH. Studies in Europe have reported that CBP housing can pose challenges in terms of achieving adequate udder health and high milk quality, especially with low space per cow [16]. In contrast, CBP systems have improved heat detection [13,17]. Differences in climate and in the management of housing systems are big sources of heterogeneity in the living conditions offered to cattle in Europe [18] and may explain the variable results.

The aim of this study was to assess cattle health in CBP and CH systems based on health indicator traits (somatic cell counts (SCC), high SCC, new high SCC, ketosis risk, prolonged calving intervals, dystocia, and stillbirth), and other traits (length of life, productive life, parity at exit from the herd, first calving risk, calf mortality) from dairy herd improvement (DHI) records collected in six European countries.

## 2. Materials and Methods

Dairy farms in Austria, Germany, Italy, the Netherlands, Slovenia, and Sweden were selected for participation in a comprehensive investigation of the animal health and welfare, milk quality, and environmental and socioeconomic impacts of using CBP and CH systems, respectively (see https://www.freewalk.eu/en/freewalk/Project.htm, accessed on 14 December 2021). Twenty CBP farms were selected: (1) to ensure that the samples were representative of the dairy production in the respective countries, with respect to herd size and milk production level; and (2) that had used CBP systems for their lactating herd for at least six months before the first farm visit, to avoid “conversion” effects and to ensure a more-or-less stable situation. Each CBP farm was matched with a CH farm within the country, according to herd size, production level, breed, and location. Some CBP herds, i.e., three in Germany and one in Italy, used the CBP for only parts of the herd, and used CH for the other parts. These herds, and their matched CH herds, were excluded from the analyses of DHI data, because such data typically refer to the whole herd and not only to the CBP parts. Thus, the statistical analyses (see below) included data from only 16 herd pairs (32 herds).

Data were retrieved retrospectively from the national DHI systems of each country. In all countries, permission was obtained from the participating farmers and database managers before data collection. The national recording systems are not harmonized; the record-keeping methods and the amount of information recorded differ. For the purpose of this study, only data available in all participating countries were used and transformed into a common structure, largely following the recommendations in the Recording Guidelines of the International Committee for Animal Recording (ICAR, http://www.icar.org/index.php/icar-recording-guidelines/, accessed on 14 December 2021).

The data retrieved contained information on animals (breed, birth date, date of entry into and exit from the herd, etc.), calvings (date, calving ease, stillbirth, etc.), inseminations/natural services, milk test days (date, milk yield, fat and protein percentages, and SCC in 1000 cells/mL), and lactation yields (milk, fat, and protein). The focus for these analyses were the years during which the herds were also visited (2017–2018), but all data in the national recording systems related to the animals in the participating farms from 2012 and onwards were requested to ensure sufficient information for relevant calculations of all traits.

The health traits considered here were:

- log_10_(SCC);
- High SCC (HiSCC; SCC >150 and >200 for first and later parities, respectively, coded 1, and 0 otherwise);
- New HiSCC, compared with previous test day (IncSCC; coded 1, and 0 otherwise);
- Risk of ketosis (KETO; defined only for test days 30–100 days after calving, with a fat/protein ratio above 1.4 coded 1, and 0 otherwise [19]);
- Prolonged calving interval (LongCI) as a proxy for reproductive health (calving interval >400 d coded 1, and 0 otherwise [20,21]);
- Dystocia (DYST; difficult calving, caesarean section, or embryotomy coded 1 and easy calving without or with some assistance coded 0; the maximum code was used when there were multiple calves born); and
- Stillbirth (STBTH; malformation, still-born (i.e., dead-born full term), or perinatal death within first 24 h coded 1, and 0 otherwise; the maximum code was used when there were multiple calves born (not calculated for herds in the Netherlands due to missing information).
- The following traits were used as indicators of culling and culling-related issues:
- Length of life (LL; days from birth to exit from the herd);
- Length of productive life (PL; days from first calving to exit from the herd) for cows with at least one calving;
- Parity at exit from the herd for lactating cows (xPAR);
- First calving risk, i.e., being a calving due to a first calver (1STC; calving number 1 coded 1, and 0 otherwise); and
- Calf mortality (death or euthanasia on farm within 30 d of birth).

The associations between housing system, i.e., CBP or CH, and the different traits defined above were assessed using multivariable (mixed) linear and logistic regression models for continuous and dichotomous outcome variables, respectively (procedures MIXED and GLIMMIX). All models included the fixed effects of the housing system, matching pair, and breed (Holstein, other), and most models also included the fixed effect of parity. Models for traits based on calvings, such as LongCI, also included the fixed effect of year of calving, while models for traits based on exits from the herd included the fixed effect of year of exit. Models for traits based on test-day observations, such as log_10_(SCC), included the fixed effect of the year–month of the test and month-in-milk class (30-d periods), and the model for calf mortality included the effect of the year of birth. A random effect of the animal nested within the herd was included in trait models in which there could be repeated observations per animal, such as log_10_(SCC). Results of the models are presented as marginal means, i.e., regression-adjusted means of the response variable. Details of the models are found in Supplementary Tables S1–S4.

Analyses of traits based on calvings used all data that pertained to calvings in 2017–2018; analyses of traits based on exits used all data on animals that exited the herds in 2017–2018; analyses of traits based on test-day observations used test days in 2017–2018; and analyses of calf mortality were based on animals born in 2017–2018.

All statistical analyses were performed with SAS/STAT 14.1 (SAS Institute Inc., Cary, NC, USA).

## 3. Results

Herd size, in terms of the average number of cows in lactation, ranged from 14 to 175; there were some notable country differences, with Austria having the smallest herds (Table 1). The average calving number in the herds was relatively uniform across the countries, but with, on average, more later parity cows in Austria and less so in Sweden (Table 1).

Most of the cows that calved in 2017 and 2018 in Austrian herds were Simmental (85%) and the rest were Holstein. The most common breed in the herds in Sweden was Swedish Red, while Holstein was the most common breed in all other countries (Table 2). The distribution of breeds was roughly the same over the housing systems, except in Austria, where almost none of the cows in CH were Holsteins, and in Germany, where almost all cows in CH were Holsteins.

Marginal means of health traits, as estimated in the multivariable regression models, for the independent variable of main interest, i.e., housing system, are presented in Table 3, while the complete list of parameter estimates and significance tests of main effects are given in Supplementary Tables S1 and S2. Cows in herds with CBP had higher SCC, both as continuous variables and in terms of the prevalence and incidence of high SCC, than did CH herds. They also had more calvings with stillbirths than did CH herds, but a smaller proportion of prolonged calving intervals.

Marginal means of traits related to culling, as estimated in the multivariable regression models, for the variable of main interest, i.e., housing system, are presented in Table 4, while the complete list of parameter estimates and significance tests of the main effects is given in Supplementary Tables S3 and S4. There was a significantly smaller proportion of first calvers in CBP herds and, therefore, a lower first calving risk.

## 4. Discussion

We found relatively small differences in health between herds with CBP and CH systems. The most obvious difference was that udder health was worse in the CBP herds. Our results are in contrast to those of other studies that found no significant difference in mastitis prevalence between systems [14,22], although Lobeck et al. [22] found a numerical difference pointing in the same direction as our results. No data on metabolic diseases were available; the fat/protein ratio was used as a proxy for ketosis risk, but, although slightly higher in CH, there was no significant difference between the housing systems. Some research comparing tie stall and CH attributed the lower rate of ketosis in CH as an effect of housing (more exercise), which could potentially be seen as an analogy to more muscle exercise in CBP [23]. Results are difficult to compare between studies, however, because of the large variations in the types of CBP and CH farms included and because of different climatic conditions and bedding management affecting the studied farms. It is conceivable, however, to find differences in udder health, because most studies have found that cows in CBP are dirtier than cows in CH [22,24,25], and cleanliness is an important factor for good udder health [26,27,28]. A properly managed CBP can provide a health-promoting, dry, and comfortable surface on which cows can lie, stand, and walk. Moreover, udder health depends on so many other aspects that were not part of the study. Feeding practices can be expected to be the same irrespective of housing system, so a similar risk of ketosis found in CBP and CH systems is conceivable. Furthermore, studies have found no difference in body condition score between CBP and CH systems [22,29], corroborating our finding. Stillbirths were more common in CBP systems, but we cannot offer any explanations for the difference. Further studies with more farms are needed with an increased use of this system. Our study sample limits the extent to which the findings can be effectively theorized and developed. It should be noted that all differences are presented as marginal means and, thus, adjusted for the other effects in the models, such as breed, which may be an important determinant of stillbirths.

The proportion of LongCIs was smaller in the CBP herds, indicating fewer problems with reproductive disturbances, and days from calving to first artificial insemination were also shorter (data not shown). Motta et al. [30] found no difference in the prevalence of follicular cysts between CBP and CH herds. It has been shown that cows in CBP have higher comfort when resting [25] and that walking might be easier in CBP [12]. In addition, lameness and hock lesion prevalences were lower in CBP than in CH [25], and lameness is a chronically painful and stressful condition associated with poor reproductive performance and reduced estrus intensity in dairy cows [31]. It is therefore plausible that cows in CBP have a better ability to express natural behavior, such as heat signs, so improved fertility is conceivable. Improved reproductive performance has also been observed in dairy herds after moving from CH to CBP [17,32]. It is unclear, however, whether the improvement was due to improved management, changes in the physiology or behavior of the animals, or a direct consequence of the housing circumstances.

There was no difference in LL, PL, or xPAR between the systems, but CBP herds had a smaller proportion of first calvers, which may indicate a lower culling rate in these herds. Some studies indicate improved longevity in CBP [16], but other studies agree with our results [22]. There are many factors underlying herd turnover rates, in relation to both management decisions and biology, and it is reasonable that this might impose considerable within-system variations, making significant systematic differences between the systems less likely.

We based our assessment of the differences in health and other cow traits only on data available through the DHI associations. The collection and management of DHI data are particular to each country, although there have been efforts to standardize such registration through the actions of ICAR. There may therefore be systematic differences in trait observations between countries, due solely to registration differences. However, utilizing the matching design (pairing CBP and CH herds within countries) in the statistical analyses of this study should have eliminated such systematic differences that affect both CBP and CH herds, so the comparisons of the two housing systems should be valid. Because of these differences in the data and the matched design, we were not able to evaluate potential differences in health between countries and, indeed, whether the differences between the housing systems varied by country. Moreover, potential differences due to other factors used in the matching, such as herd size, milk production, and breed, could not be evaluated. Data availability also imposed limitations on assessing potential differences in several economically important health traits, such as clinical mastitis and lameness. Further studies are therefore needed, probably using primary rather than secondary data, to explore the potential effects of geographic or climatic differences on health in the two housing systems.

It is likely that the housing system has long-term effects on cow health and longevity, and that the effects of a given system on certain traits cannot be seen in the short run. The age of the system would therefore be relevant to control for in analyses such as ours. Most CBP herds had used their systems for under five years when the project started. However, years in use was almost completely confounded by housing system and could therefore not be included in the statistical analyses. Further analyses of DHI data are therefore warranted when the CBP herds have had their housing systems in use for longer.

## 5. Conclusions

We found relatively small differences in health between herds with CBP and CH systems. The most obvious difference was that udder health was worse in the CBP systems. We conclude that more research is needed to capture the long-term effects of CBP systems on dairy cattle health and longevity.

## Figures and Tables

**Table 1:** Mean (min–max) average number of cows in lactation and calving number per herd and year, in 2017 and 2018, according to country and housing system.

Country	Number of Farms	Compost-Bedded Pack		Cubicle	
		Number of Cows in Lactation	Calving Number	Number of Cows in Lactation	Calving Number
Austria	6	29.3 (14.1–40.5)	3.1 (2.8–3.2)	42.7 (27.8–71.9)	3.3 (3.0–3.7)
Germany	6	85.3 (63.8–110.8)	2.6 (2.4–2.9)	90.5 (59.0–113.2)	2.7 (2.6–2.8)
Italy	6	54 (43.2–71.5)	3.2 (2.8–3.9)	52.2 (48.1–58.3)	3.1 (2.6–3.4)
Netherlands	10	111.7 (84.9–174.6)	2.9 (2.1–3.2)	112.8 (89.4–156.2)	2.7 (2.3–3.3)
Slovenia	2	52.1	2.7	63.8	2.3
Sweden	2	90.9	2.3	105.4	2.2

**Table 2:** Distribution of cow breeds among cows that calved in 2017 or 2018.

Country	Breed						*N* ^1^
	Holstein	Rossa Reggiana	Simmental	Swedish Red	Crosses	Other	
Austria	15%		85%				556
Germany	90%				7%	3%	1374
Italy	65%	14%			21%		800
Netherlands	78%				22%		2255
Slovenia	100%						267
Sweden	11%			51%	38%		514
Average	68%	2%	8%	5%	17%	0%	5766

^1^*N* = total number of calvings.

**Table 3:** Marginal means (95% confidence intervals [CIs]) of health traits per housing system estimated from mixed linear and logistic regression models, and significance test of differences between housing systems.

Trait	Compost-Bedded Pack		Cubicle		*p*-Value
	Marginal Mean	No. Cows	Marginal Mean	No. Cows	
Somatic cell count (SCC) in 1000 cells/mL ^1^	86.9 (83.2–90.7)	2039	67.7 (64.9–70.7)	2288	<0.001
High SCC, percent ^2^	18.9 (17.6–20.3)	2039	12.9 (11.9–13.9)	2288	<0.001
New high SCC, percent ^2^	12.2 (10.6–13.7)	1846	8.2 (7.1–9.3)	2097	<0.001
Ketosis risk, percent ^2^	9.8 (8.7–10.9)	1789	10.3 (9.2–11.4)	2016	0.44
Prolonged calving intervals, percent ^2^	34.7 (32.1–37.2)	1319	39.8 (37.1–42.4)	1525	<0.01
Dystocia, percent ^2,3^	2.7 (2.0–3.3)	1802	3.3 (2.5–4.0)	2115	0.16
Stillbirth, percent ^2,4^	3.9 (2.8–5.0)	1080	2.5 (1.7–3.2)	1339	<0.01

^1^ Marginal means, and CIs, were calculated using a log10 scale and transformed to the observed scale, i.e., the equivalent to geometric means. ^2^ Presented as predicted probabilities. ^3^ Some pairs had no dystocia and the model did not converge, so “pair” was excluded from the model. ^4^ Model with random effect of cows did not converge, so the effect was excluded.

**Table 4:** Marginal means (95% confidence intervals) of traits related to culling per housing system estimated from mixed linear and logistic regression models, and significance test of difference between housing systems.

Trait	Compost-Bedded Pack		Cubicle		*p*-Value
	Marginal Mean	No. Cows	Marginal Mean	No. Cows	
Length of life, months	68.4 (65.5–71.2)	521	67.8 (65.3–70.3)	728	0.72
Productive life, months	33.7 (31.0–36.4)	476	33.7 (31.2–36.2)	698	1.0
Parity at exit from the herd, parity number	3.48 (3.28–3.68)	521	3.62 (3.43–3.80)	728	0.26
First calving risk, percent ^1^	26.2 (24.3–28.0)	1819	29.8 (27.9–31.7)	2150	<0.01
Calf mortality, percent ^1^	0.104 (0.071–0.136)	2452	0.094 (0.066–0.122)	2775	0.49

^1^ Presented as predicted probabilities.

## Data Availability

The data are not publicly available due to confidentiality agreements with the national data providers.

## Supplementary Materials

The following are available online at https://www.mdpi.com/article/10.3390/ani12030396/s1, Table S1: parameter estimates (Est.) with standard errors (SE) and overall significance tests of main effects from multivariable (mixed) regression models on health traits (log_10_ somatic cell counts (SCC, log_10_(SCC)), high SCC (HiSCC), new HiSCC (IncSCC), risk of ketosis (KETO)), Table S2: parameter estimates (Est.) with standard errors (SE) and overall significance tests of main effects from multivariable (mixed) regression models of health traits (prolonged calving intervals (LongCI), dystocia [DYST], stillbirth [STBTH]), Table S3: parameter estimates (Est.) with standard errors (SE) and overall significance tests of main effects from multivariable linear regression models of culling and culling-related issues (length of life, length of productive life, parity at culling), Table S4: parameter estimates (Est.) with standard errors (SE) and overall significance tests of main effects from multivariable (mixed) regression models of culling, and culling-related issues (first calving risk, calf mortality).
