# Supplementary material for: Animal Health in Compost-Bedded Pack and Cubicle Dairy Barns in Six European Countries
Source: Animals (Basel). 2022 Feb 7;12(3):396. doi: 10.3390/ani12030396 (PMC8833726; doi:10.3390/ani12030396)
Supplement: Supplementary file 1 [file animals-12-00396-s001.zip › animals-1533592-supplementary.pdf]

Table S1. Parameter estimates (Est.) with standard errors (SE) and overall significance tests of main effects from multivariable (mixed) regression models on health traits ($\log_{10}$ somatic cell counts [SCC, $\log_{10}$SCC], high SCC [HiSCC], new HiSCC [IncSCC], risk of ketosis [KETO]).

		**Log_{10}SCC**			**HiSCC**			**IncSCC**			**KETO**		
**Effect**		**Est.**	**SE**	**P-value for main effect**	**Est.**	**SE**	**P-value for main effect**	**Est.**	**SE**	**P-value for main effect**	**Est.**	**SE**	**P-value for main effect**
Intercept		2.02	0.03		-1.22	0.17		-2.12	0.80		-1.36	0.26	
Pair	1	-0.15	0.03	<0.0001	-0.23	0.15	<0.0001	-0.37	0.14	<0.0001	0.17	0.22	<0.0001
	2	-0.29	0.03		-0.87	0.14		-0.93	0.13		0.60	0.20	
	3	-0.14	0.03		-0.46	0.15		-0.83	0.14		0.15	0.22	
	4	-0.23	0.03		-0.85	0.15		-1.09	0.14		0.34	0.20	
	5	-0.34	0.03		-1.02	0.15		-0.92	0.14		-0.29	0.22	
	6	-0.07	0.03		-0.09	0.14		-0.26	0.13		0.28	0.20	
	7	0.00	.		0.00	.		0.00	.		0.00	.	
	11	0.21	0.03		0.77	0.14		0.57	0.13		0.54	0.21	
	12	-0.07	0.04		-0.36	0.18		-0.39	0.16		-1.17	0.31	
	14	-0.07	0.04		0.17	0.16		-0.18	0.15		1.26	0.21	
	15	-0.08	0.04		-0.19	0.17		-0.30	0.16		-0.25	0.27	
	16	-0.58	0.05		-2.05	0.26		-2.18	0.27		-0.26	0.30	
	17	-0.20	0.05		-0.59	0.25		-0.52	0.23		0.49	0.31	
	18	-0.16	0.04		-0.39	0.18		-0.10	0.16		-0.04	0.25	
	19	-0.15	0.04		-0.34	0.17		-0.76	0.16		1.28	0.21	
	20	0.13	0.04		0.33	0.15		0.08	0.14		0.20	0.22	
Housing system	Compost-bedded pack	0.11	0.01	<0.0001	0.46	0.05	<0.0001	0.44	0.05	<0.0001	-0.05	0.07	0.44
	Cubicle	0.00	.		0.00	.		0.00	.		0.00	.	
Month in milk class	1	0.00	.		0.00	.		0.00	.				
	2	-0.15	0.01	<0.0001	-0.36	0.06	<0.0001	0.27	0.78	<0.0001	-0.80	0.06	<0.0001

		3	-0.12	0.01		-0.33	0.06		0.25	0.78		-1.12	0.10	
		4	-0.08	0.01		-0.26	0.06		0.25	0.78		0.00	.	
		5	-0.04	0.01		-0.17	0.06		0.33	0.78				
		6	-0.01	0.01		-0.11	0.06		0.34	0.78				
		7	0.04	0.01		0.03	0.06		0.46	0.78				
		8	0.08	0.01		0.16	0.06		0.61	0.78				
		9	0.11	0.01		0.19	0.06		0.55	0.78				
		10	0.15	0.01		0.36	0.06		0.77	0.78				
		11	0.19	0.01		0.31	0.07		0.70	0.78				
		12	0.20	0.01		0.29	0.08		0.79	0.78				
		13	0.25	0.03		0.30	0.17		0.81	0.81				
Parity		1	-0.23	0.01	<0.0001	-0.75	0.05	<0.0001	-0.58	0.05	<0.0001	-0.39	0.08	<0.0001
		2	-0.11	0.01		-0.53	0.04		-0.42	0.05		-0.46	0.08	
		≥3	0.00	.		0.00	.		0.00	.		0.00	.	
Breed	Holstein		0.04	0.02	<0.01	0.19	0.07	0.01	0.15	0.07	0.03	-0.17	0.10	0.09
	Other		0.00	.		0.00	.		0.00	.		0.00	.	
Year-month of test		201701	-0.01	0.02	<0.01	-0.02	0.10	<0.001	-0.11	0.14	0.56	0.27	0.20	<0.0001
		201702	-0.01	0.02		0.06	0.10		0.07	0.14		-0.03	0.22	
		201703	-0.03	0.02		-0.03	0.09		-0.14	0.14		0.17	0.21	
		201704	-0.03	0.02		-0.08	0.10		-0.06	0.14		0.34	0.22	
		201705	-0.04	0.02		-0.14	0.10		-0.03	0.13		-0.11	0.22	
		201706	0.00	0.02		0.17	0.10		0.10	0.14		0.03	0.23	
		201707	0.00	0.02		0.00	0.10		-0.12	0.14		0.18	0.23	
		201708	0.00	0.02		0.07	0.10		0.13	0.14		0.25	0.21	
		201709	-0.01	0.02		-0.11	0.10		0.10	0.14		-0.14	0.22	
		201710	-0.03	0.02		-0.12	0.10		-0.05	0.14		-0.17	0.22	
		201711	0.00	0.02		0.05	0.10		0.11	0.14		-0.58	0.23	

201712	-0.02	0.02	-0.14	0.10	-0.05	0.14	0.01	0.21
201801	-0.02	0.02	-0.04	0.09	0.01	0.14	0.22	0.21
201802	-0.01	0.02	-0.15	0.11	0.04	0.15	0.13	0.24
201803	-0.01	0.02	-0.08	0.10	0.07	0.14	0.32	0.21
201804	0.00	0.02	0.01	0.10	0.05	0.14	0.36	0.21
201805	-0.01	0.02	-0.11	0.10	-0.12	0.15	-0.24	0.25
201806	0.00	0.02	0.08	0.10	-0.01	0.14	-0.46	0.24
201807	0.03	0.02	0.17	0.09	0.11	0.14	-0.05	0.22
201808	-0.01	0.02	0.15	0.10	0.13	0.14	-0.20	0.23
201809	-0.01	0.02	0.09	0.10	-0.05	0.14	0.21	0.21
201810	-0.01	0.02	0.05	0.10	0.02	0.14	-0.38	0.22
201811	-0.02	0.02	-0.07	0.10	-0.14	0.14	-0.02	0.21
201812	0.00	.	0.00	.	0.00	.	0.00	.

Table S2. Parameter estimates (Est.) with standard errors (SE) and overall significance tests of main effects from multivariable (mixed) regression models of health traits (prolonged calving intervals [longCI], dystocia [DYST], stillbirth [STBTH]).

		longCI			DYST			STBTH		
**Effect**		**Est.**	**SE**	**P-value for main effect**	**Est.**	**SE**	**P-value for main effect**	**Est.**	**SE**	**P-value for main effect**
Intercept		-0.27	0.18		-3.87	0.21		-4.98	0.50	
Pair	1	-0.33	0.20	<0.0001						
	2	-0.16	0.18							
	3	1.00	0.22							
	4	-0.30	0.19							
	5	0.06	0.19							
	6	-0.23	0.18					0.99	0.43	0.01
	7	0.00	.					0.00	.	
	11	-0.02	0.19					1.18	0.43	
	12	-0.03	0.22					0.76	0.57	
	14	0.06	0.21					-0.22	0.60	
	15	-0.56	0.23					0.57	0.53	
	16	-0.45	0.27					1.42	0.63	
	17	-0.91	0.33					0.73	0.86	
	18	-0.67	0.23					1.21	0.50	
	19	0.35	0.21					1.45	0.44	
	20	-0.09	0.21					1.15	0.50	
Housing system	Compost-bedded pack	-0.22	0.07	<0.01	-0.21	0.15	0.16	0.47	0.17	<0.01
	Cubicle	0.00	.		0.00	.		0.00	.	
Parity	1	-0.22	0.07	<0.01	0.29	0.17	0.15	0.52	0.19	<0.01

		2	0.00	.		-0.02	0.19		-0.28	0.25	
		≥3	0.17	0.10		0.00	.		0.00	.	
Breed	Holstein		0.00	.	0.1	0.62	0.18	<0.001	0.92	0.28	<0.001
	Other					0.00	.		0.00	.	
Year of calving		2017	0.05	0.07	0.44	0.16	0.15	0.26	-0.14	0.17	0.4
		2018	0.00	.		0.00	.		0.00	.	

Table S3. Parameter estimates (Est.) with standard errors (SE) and overall significance tests of main effects from multivariable linear regression models of culling and culling-related issues (length of life, length of productive life, parity at culling).

Effect		Length of life			Productive life			Parity at culling		
		Est.	SE	P-value for main effect	Est.	SE	P-value for main effect	Est.	SE	P-value for main effect
Intercept		1465.10	111.27		636.26	98.53		2.53	0.26	
Pair	1	259.67	113.82	<0.0001	380.91	98.08	<0.0001	0.49	0.27	<0.0001
	2	1110.09	131.85		1244.31	114.21		2.50	0.31	
	3	430.44	112.81		532.19	97.12		0.23	0.27	
	4	711.80	106.43		820.87	91.51		1.48	0.25	
	5	492.60	117.73		539.85	103.11		0.88	0.28	
	6	146.45	111.83		174.61	94.12		0.14	0.27	
	7	0.00			0.00			0.00		
	11	229.60	104.69		252.04	88.70		0.45	0.25	
	12	1234.31	155.89		233.65	166.36		2.03	0.37	
	14	544.00	116.91		248.98	107.82		1.11	0.28	
	15	418.48	130.47		347.03	117.90		0.89	0.31	
	16	1020.73	194.74		485.74	198.24		2.24	0.46	
	17	996.28	242.79		131.55	287.61		2.30	0.58	
	18	429.80	137.79		153.19	129.33		0.68	0.33	

	19	442.80	120.46		248.48	109.95		0.81	0.29	
	20	271.88	117.33		160.66	105.49		0.21	0.28	
Housing system	Compost-bedded pack	18.23	50.10	0.72	0.21	45.68	1	-0.14	0.12	0.26
	Cubicle	0.00	.		0.00	.		0.00	.	
Breed	Holstein	100.40	83.51	0.23	100.30	76.78	0.19	0.07	0.20	0.71
	Other	0.00	.		0.00	.		0.00	.	
Year of culling	2017	10.5815	48.2626	0.82	-59.3316	43.957	0.18	0.03829	0.1148	0.74
	2018	0	.		0	.		0	.	

Table S4. Parameter estimates (Est.) with standard errors (SE) and overall significance tests of main effects from multivariable (mixed) regression models of culling and culling-related issues (first calving risk, calf mortality).

Effect		% 1st calvers			Calf mortality		
		Est.	SE	P-value for main effect	Est.	SE	P-value for main effect
Intercept		-0.38	0.15		-3.42	0.42	
Pair	1	-0.29	0.16	<0.0001	0.44	0.45	<0.0001
	2	-0.46	0.15		0.68	0.42	
	3	-0.31	0.18		0.50	0.46	
	4	-0.57	0.16		0.23	0.45	
	5	-0.40	0.16		1.13	0.42	
	6	-0.27	0.15		0.55	0.46	
	7	0.00			0.00		
	11	-0.22	0.15		-0.22	0.51	
	12	-0.64	0.19		-15.13	0.00	
	14	-0.57	0.18		-15.20	0.00	
	15	-0.42	0.19		-15.19	0.00	
	16	-0.86	0.24		0.54	0.53	
	17	-0.63	0.26		-0.19	0.71	
	18	-0.51	0.19		1.07	0.44	

	19	-0.12	0.17		-15.24	0.00	
	20	0.00	0.16		-0.45	0.52	
Housing system	Compost-bedded pack	-0.18	0.06	0.003	0.10	0.14	0.49
	Cubicle	0.00	.		0.00	.	
Breed	Holstein	-0.07	0.09	0.45	0.15	0.15	0.33
	Other	0.00	.		0.00	.	
Year of culling	2017	-0.1041	0.05865	0.08	-0.1806	0.1377	0.19
	2018	0	.		0	.	
